# Sterile syringe access and disposal among injection drug users newly enrolled in methadone maintenance treatment: a cross-sectional survey

**DOI:** 10.1186/1477-7517-3-8

**Published:** 2006-02-18

**Authors:** Jennifer McNeely, Julia H Arnsten, Marc N Gourevitch

**Affiliations:** 1 Department of Medicine, Brigham and Women's Hospital, Boston, MA, USA; 2 Department of Psychiatry and Behavioral Sciences, Albert Einstein College of Medicine, Bronx, NY, USA; 3 Division of General Internal Medicine, Department of Medicine, Albert Einstein College of Medicine and Montefiore Medical Center, Bronx, NY, USA; 4 Department of Epidemiology and Population Health, Albert Einstein College of Medicine and Montefiore Medical Center, Bronx, NY, USA; 5 Division of General Internal Medicine, Department of Medicine, New York University School of Medicine, New York, NY, USA

## Abstract

**Background:**

We sought to assess injection practices, means of acquiring and disposing of syringes, and utilization and knowledge of harm reduction resources among injection drug users (IDUs) entering methadone maintenance treatment (MMT).

**Methods:**

Interviews with 100 consecutive patients, including 35 IDUs, entering a MMT program in the Bronx, NY.

**Results:**

Utilization of unsafe syringe sources was reported by 69% of IDUs in our sample. Most (80%) IDUs reused syringes, and syringe sharing was also common. Fewer than half knew that non-prescription pharmacy purchase of syringes was possible. The most common means of disposing of injecting equipment were the trash (63%) and syringe exchange programs (49%).

**Conclusions:**

These findings indicate that drug users entering treatment under-utilize sanctioned venues to obtain sterile syringes or safely dispose of used injection equipment. Programs providing services to drug users should adopt a proactive stance to address the safety and health issues faced by injectors.

## Introduction

The need for injection drug users (IDUs) to have access to sterile syringes for the prevention of blood-borne disease and other health problems is well established [1]. Injection drug use accounts for approximately one-third of all AIDS cases in the United States, and for 60% of new hepatitis C infections [2]. The Institute of Medicine of the National Academy of Sciences has stated that " [f]or injection drug users who cannot or will not stop injecting drugs, the once-only use of sterile needles and syringes remains the safest, most effective approach for limiting HIV transmission" [3]. Nonetheless, IDUs' access to sterile syringes is generally inadequate to limit their risk of acquiring and transmitting infections [4-6]

Syringe exchange is associated with reduced HIV transmission among IDUs [7,8], yet existing syringe exchange programs (SEPs) are able to meet only a fraction of injectors' need for sterile injection equipment [4]. To increase access to sterile syringes, New York State enacted the Expanded Syringe Access Program (ESAP) in 2001, which legalized pharmacy sale of syringes to adults without a prescription. This program provides a means for drug treatment and other health care providers to promote safer injecting practices to their drug using patients without providing syringes on-site. Yet ESAP, while a significant step forward, has been utilized only modestly, due in part to a ban on advertising and to slow uptake by service providers working with drug users [9].

Methadone maintenance treatment (MMT), because of its effectiveness in treating heroin dependence, is also associated with reduced risk for acquiring or transmitting HIV infection [10-12]. Yet while the majority of patients receiving methadone maintenance treatment cease using heroin, some continue to inject, particularly in the early months of treatment [13]. Others inject cocaine, a behavior that methadone influences minimally if at all [14,15].

Education about safer drug use ("harm reduction") and related interventions, including provision of sterile syringes, have not been accepted practice at most methadone maintenance treatment programs (MMTPs) in the United States. This reflects, in part, the highly regulated model of MMT that evolved in the United States well before the age of HIV/AIDS, as well as the strong focus on abstinence of many MMTPs [16,17]. The notion of offering methadone treatment along with access to sterile syringes seems discordant, or even contradictory, to many MMT staff. Finally, some MMT providers are concerned that acknowledgment of ongoing drug use among their patients might damage already shaky community and public relations. Together, these obstacles have hindered MMTPs from endorsing harm reduction interventions such as access to sterile injection equipment.

To determine the need and acceptability for an intervention at a MMT to facilitate sterile syringe access and safe disposal, we surveyed patients newly enrolled in methadone maintenance treatment to define their injecting behaviors and to assess their access to sterile syringes and their syringe disposal practices.

## Methods

This study was conducted at a single MMTP in the Bronx, NY, which serves as the intake site for a network of 8 additional MMTPs. Patients undergo comprehensive medical and psychosocial assessment and methadone induction, typically receiving care at this clinic for six months and attaining a stable methadone dose before being transferred to another site in the system for ongoing maintenance treatment. The average census of the intake clinic is 500 patients.

A 24-item survey was administered to all consecutive new entrants to the MMTP over a 3-month period in 2002–2003, beginning 22 months after ESAP's implementation. Study eligibility criteria specified that, in addition to meeting the program's entry criteria (≥18 years old; ≥1 year of opioid dependence; and at least one prior attempt at treatment or detoxification), participants must have been in treatment for more than 2 weeks and less than 2 months. To ensure that all study participants were just beginning methadone treatment, patients were excluded if they had transferred directly from another MMTP without a lapse in methadone treatment.

Patients were approached in the waiting area and asked to participate in the survey. Participation was voluntary, and no compensation was offered. The survey was administered in English or Spanish by one of us (JM), who read the questions and recorded patient responses. All information was self-reported. None of the responses were shared with the MMTP staff, and the interviewer was not part of the treatment team. Verbal informed consent was obtained from all participants, and the study was approved by the institutional review board of the Albert Einstein College of Medicine.

The survey included questions about injection history, sources of syringes, knowledge of and experiences with the ESAP program, methods of disposing of used injecting equipment, and self-reported viral hepatitis and HIV infection status. Subjects were asked to name all places at which they obtained syringes in the past 6 months, to specify their primary source of syringes, and to identify all methods used to dispose of used syringes in the past year. Participants' opinions as to whether MMTPs should distribute syringes or have sharps containers on-site were also solicited.

Patients who reported injecting drugs during the prior six months were designated "current IDUs" and were selected for analysis. Needle or syringe exchange programs, pharmacies, and medical providers were classified as "safe" sources of syringes. Street sellers, acquaintances (friend, relative, partner, spouse), shooting galleries, or needles found on the street were considered "unsafe" sources, since the sterility of syringes obtained from these sources is not assured. Frequency analyses were performed using SPSS (SPSS for Windows, Chicago, IL, 1999).

## Results

Of 108 patients meeting eligibility criteria, 4 (4%) left the program before being interviewed, and 4 (4%) declined to participate. Of the remaining 100 participants, 35 were current IDUs, and are the subject of this analysis.

Study participants had a mean age of 38 years (range 19–61), and the majority was male (Table 1). Seventy-four percent were Hispanic and 14% African American. Self-reported prevalence of HIV infection was 31%, and hepatitis C infection was reported by 69%. The majority of subjects (94%) were interviewed during the 3rd or 4th week of their participation in methadone treatment. Five interviews (14%) were administered in Spanish.

**Table 1:** Patient Demographics: Recent MMTP entrants who injected drugs in the past 6 months

**Characteristic**	**Participants (n = 35)** **No.(%)**
**Age**,	
mean (range)	38 (19–61)
**Sex**	
Men	19 (54.3)
**Ethnicity**	
Hispanic	26 (74.3)
African American	5 (14.3)
Caucasian	4 (11.4)
HIV positive*	11 (31.4)
HCV positive*	24 (68.6)
**Time in treatment**	
2–4 weeks	33 (94.3)
5–8 weeks	2 (0.6)
**Recent injection drug use**	
Injected in past week	18 (51.4%)
Injected in past month	28 (80.0%)

*Self-reported

Among participants reporting injecting during the previous six months, 51% (n = 18) had injected in the past week, and an additional 29% (n = 10) had injected in the past month, despite being enrolled in methadone treatment for the past several weeks. An additional 23 participants (from the original sample of 100) had a history of injection drug use, but had not injected in the past six months.

Current IDUs' sources of syringes during the prior 6 months are presented in Table 2. Though half (53%) used a primary syringe source that was "safe," utilization of unsafe sources was common. The majority (69%) of IDUs reported some utilization of unsafe sources, predominantly street sellers (including drug dealers and needle sellers). Friends, relatives, spouses, and partners were also commonly cited sources. Syringe exchange programs were heavily utilized, with 46% of participants reporting obtaining syringes from an exchange, and 40% citing exchanges as their primary syringe source. Pharmacies were a primary syringe source for only 11% (n = 4), but were utilized at least occasionally by an additional 20% (n = 7). During the past six months, 2 subjects reported having visited a shooting gallery, and 1 had used a syringe found on the street. None of these current IDUs had received syringes from a health care provider.

**Table 2:** Sources of syringes in the past 6 months (n = 35)

**Source**	**Utilized ** **No. (%)**	**Primary source** **No. (%)**
Safe Sources	22 (62.9)	18 (52.9)
Syringe exchange program	19 (45.7)	14 (40.0)
Pharmacy	11 (31.4)	4 (11.4)
Unsafe Sources	24 (68.6)	16 (47.0)
Purchased on street	18 (51.4)	8 (22.9)
Friends/relatives	15 (42.9)	7 (20.0)
Spouse/partner	10 (28.6)	1 (2.9)
Shooting gallery	2 (5.7)	0
Found	1 (2.9)	0

Measures of syringe access and availability are shown in Table 3. Most (80%) subjects stated that they typically reused syringes, and syringe sharing was common. Only 46% of these current IDUs knew that it was possible to buy syringes at a pharmacy without a prescription, and 43% (n = 15) had attempted to do so. Among those who tried buying syringes in a pharmacy, half reported having been refused sale on at least one occasion.

**Table 3:** Measures of syringe access and availability

**Question**	**Participants (n = 35)** **No. (%)**
How many times do you usually use a syringe?	
1	7 (20.0)
2–5	23 (65.7)
>5	5 (14.3)
Have you shared syringes in the past 6 months?	
Yes	14 (40.0)
No	21 (60.0)
How much do you usually pay for a syringe?	
$0	16 (45.7)
< $1	7 (20.0)
$1	4 (11.4)
$2	8 (22.8)
Have you ever used a needle exchange program?	
Yes	22 (62.9)
No	13 (37.1)
Are you aware of ESAP, or do you know that adults can buy syringes in the pharmacy without a prescription?	
Yes	16 (45.7)
No	19 (54.3)
Have you tried to purchase syringes in pharmacy since January 2001?	
Yes	15 (42.9)
No	20 (57.1)
Have you tried to buy syringes in a pharmacy and been refused since January 2001?	
Yes	7 (50.0% of those attempting purchase)
No	7 (50.0% of those attempting purchase)

More than half (54%) of participants paid for syringes, at prices ranging up to two dollars for a single syringe. Those who reported pharmacies as their primary source of syringes paid less than one dollar per syringe (modal price less than $0.50), while those who bought syringes primarily from street sellers paid fifty cents to two dollars per syringe (modal price $2.00). Of those who named syringe exchange programs as their primary source of syringes, 38% (n = 5) typically traveled more than 20 minutes (or >10 blocks) to get syringes. No subject who named either pharmacies or street sellers as their primary source of syringes traveled this far.

**Figure 1:**
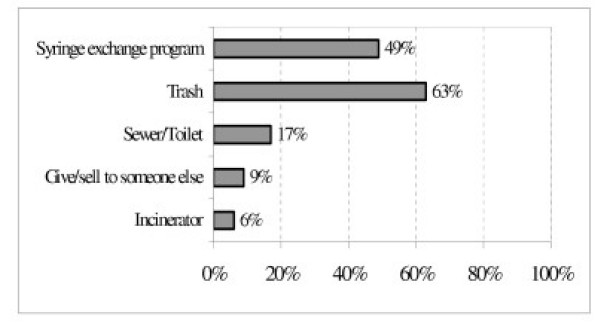
Location of syringe disposal (n = 35).

Means of disposal of used syringes are shown in Figure 1. By far the most common disposal site was the regular trash. Most participants reported breaking the syringe tip before discarding, and 32% of those disposing in the trash reported placing their syringes in a container before throwing them away. The second most common means of disposal was the sharps container at a syringe exchange program, utilized by 49% of participants.

## Discussion

Recent enrollees to MMT who continue to inject drugs often utilize unsafe means of obtaining and disposing of syringes. Though MMT is effective at reducing and eliminating heroin use, some patients continue to inject illicit drugs during treatment [13-15]. Because of their emphasis on eliminating drug use, MMTPs traditionally have not focused on harm reduction, nor explicitly supported safer injection practices among patients who continue to inject. Yet elimination of drug use is not an immediately attainable goal for many patients, whose risk of acquiring and transmitting infections and of other injection-related harm persists despite participation in drug treatment. Our data make clear the need for increased attention to safer injecting practices for IDUs recently enrolled in MMTP, and suggest that treatment programs should play a more proactive role in promoting safe syringe use and disposal.

Our data suggest that even for IDUs residing in an urban setting rich in syringe exchange programs, with over 100 pharmacies registered to sell syringes, access to safe syringes and proper disposal is inadequate. Forty percent (40%) of IDUs reported sharing syringes in the past 6 months, and only one in five was able to use a new syringe for each injection. Though very high-risk sources such as shooting galleries and found syringes were avoided by most participants, even low-level use of such sources is highly problematic, given the considerable risk of disease transmission. The rate of syringe sharing in our sample is similar to that reported in a study of IDUs in New York City in 1996–1997, which documented "receptive" and "distributive" sharing rates of 24% and 19%, respectively [18]. Though substantial geographic and temporal variation exists in drug injectors' access to SEPs and other sources of sterile injection equipment in the United States [19-26], our data are consistent with the findings of investigators in a range of locations that efforts to ensure adequate community-level access are generally insufficient to meet the demand [3-6].

Over-the-counter syringe purchase in pharmacies (the ESAP program) has been an important initiative to increase access to safe injection equipment for injectors in New York State. The fact that many of the injectors in our sample had used pharmacies to buy syringes is testimony to the success of the ESAP program. Further work remains, however, to optimize its impact. Though ESAP was implemented almost two years prior to our interviews, most IDUs were not aware of its essential benefit; that they could purchase syringes at pharmacies without a prescription. Utilization rates in our treatment population are not dissimilar from those reported among IDUs in Harlem and the South Bronx, according to an independent evaluation of the ESAP program [9]. This evaluation also suggested that African American and Hispanic IDUs (the majority of our study population) are much less likely to report pharmacy purchase. Significantly greater community outreach regarding the ESAP program is clearly indicated.

Pharmacy refusal to sell syringes was commonly experienced by study participants. While 43% of our participants had attempted to purchase syringes at a pharmacy since the implementation of ESAP, only 31% reported succeeding. Refusals to sell syringes were documented in the first year of ESAP by Finkelstein et al., who found that 31% of New York City pharmacists and 67% of those in the Bronx declined to sell syringes despite being registered ESAP providers [27].

Syringe exchange programs were a major source of syringes for our participants, but IDUs who relied on them as the primary source of syringes reported traveling much farther to get syringes than those who used other sources. SEPs also have limited hours for syringe exchange, which may be an additional hardship, relative to pharmacy sale, for some users. Participants who utilized syringe exchange programs were more likely to use them as their primary source of syringes, while pharmacy purchasers were more likely to use the pharmacy as a secondary or supplemental source. This might reflect the fact that syringes are free at syringe exchange programs, and that IDUs are taking advantage of the other services (meals, caseworkers, support groups, acupuncture, etc.) offered at SEPs. It may also be that IDUs have other reasons to feel less comfortable purchasing syringes in a pharmacy, including reluctance to reveal themselves as drug users, fear of discrimination, and concerns regarding stigmatization or refusal of sale by the pharmacist.

Our finding that regular trash is the most commonly cited syringe disposal site points starkly to the need for improved systems for safe syringe disposal. Disincentives for IDUs to carry syringes to a safer disposal location include fear of arrest or harassment by the police, and of being identified as a drug user [28,29]. Yet nearly half of current IDUs reported disposing of syringes at a syringe exchange program at least once in the past year, suggesting that innovative strategies for community-based disposal may have positive results.

Our study has several limitations. The size of our sample of current IDUs was modest. The proportion of IDUs in our sample of consecutive treatment entrants is consistent with current trends in New York City [30]. We sampled participants at a single South Bronx MMTP. However, since this research site is the intake program for a large network of methadone programs, it draws patients from throughout the Bronx and, to a lesser extent, other areas of New York City. This MMTP thus has a high proportion of patients in their first months of methadone treatment, a subgroup for whom our findings are especially applicable. Selection bias was minimized by enrolling consecutive patients and by the high response rate. Although we used self-report data, and thus may have underestimated the prevalence of disease and of injecting and other risky behaviors, other studies of IDUs have demonstrated good reliability using similar questions [31,32].

Our findings suggest that drug treatment programs serving injectors should more actively address the safety and health issues associated with injection drug use. Thoughtful education of patients, staff and communities may be needed to help drug treatment and harm reduction providers recognize their common ground. As a significant number of patients continue to inject during the early phase of methadone maintenance treatment, injectors receiving services in this setting should not be excluded from complementary efforts to minimize injection-related harm.

To address these issues successfully, drug treatment programs might offer education, sterile syringe access, and syringe disposal through a combination of on-site services and referral to community-based providers. By legalizing pharmacy purchase of syringes, ESAP and similar programs provide an opportunity for providers of drug treatment and health care services to promote health-protecting behaviors to patients who continue to inject. Innovative strategies to enhance access to sterile syringes and foster safe syringe disposal for MMTP participants who continue to inject merit vigorous exploration.

## Competing interests

The author(s) declare that they have no competing interests.
